# Dorsal Genital Nerve Stimulation as an Adjunctive Therapy to Control Neurogenic Detrusor Overactivity After Spinal Cord Injury

**DOI:** 10.1111/aor.15026

**Published:** 2025-05-26

**Authors:** Sean Doherty, Evangeline Martinez, Sarah Knight, Richard Nobrega, Lynsey Duffell

**Affiliations:** ^1^ Department of Medical Physics & Biomedical Engineering University College London England UK; ^2^ London Spinal Cord Injury Centre Royal National Orthopaedic Hospital Stanmore England UK

**Keywords:** dorsal genital nerve stimulation, neurogenic detrusor Overactivity, spinal cord injury

## Abstract

**Background:**

Spinal cord injury (SCI) causes impairment to bladder function. As current management strategies are not fully effective, there remains a need for alternative or adjunctive management options. Dorsal genital nerve stimulation (DGNS) has been shown to effectively inhibit unwanted bladder contractions in people with SCI. However, the acceptance and beneficial effects of home‐based DGNS have not yet been proven.

**Methods:**

This pilot trial investigated the feasibility and user acceptance of a novel DGNS device, UCon (InnoCon Medical, Denmark) when used at home by people with SCI for 8 weeks. Cystometry with and without DGNS was performed before and after the intervention. Outcome measures included 3‐day bladder diaries (3DBD), quality of life, and user acceptance questionnaires, and a semi‐structured interview.

**Results:**

Ten people with chronic SCI and neurogenic detrusor overactivity participated in the trial and used the device at home for 8 weeks. When tested at baseline, DGNS significantly increased maximum cystometric capacity (MCC) from 276 (125) to 394 (147) ml with DGNS applied (*p* < 0.001). After the 8‐week intervention, no change in MCC without stimulation was noted. No change in bladder capacity or incontinence episodes was found from 3DBDs and quality of life was not significantly changed. User acceptance of the device was high, with some issues noted, particularly around device disconnection.

**Conclusions:**

Our results support the application of DGNS to increase bladder capacity after SCI, and user acceptance of home‐based DGNS was high. This therapy should be studied in a larger subject group to prove the effectiveness of home‐based DGNS.

## Graphical abstract

People with spinal cord injury (SCI) experience bladder overactivity, which can cause incontinence. Dorsal genital nerve stimulation (DGNS) can suppress overactivity, providing a longer duration to reach a toilet. People with SCI trialed home‐based DGNS for 8 weeks. DGNS was effective in the laboratory, and participants found home‐based DGNS acceptable.

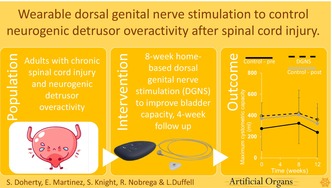

## 1 Introduction

In the United Kingdom, there are approximately 105 000 people living with SCI, with 4400 new cases each year [1]. Restoration of pelvic functions remains one of the top priorities for people with spinal cord injury [2, 3]. A Priority Setting Partnership by the James Lind Alliance and Spinal Cord Injured charities has published the top 10 research priorities of patients with a spinal cord injury. Bladder and bowel management was mentioned in three of these priorities [4].

Bladder and bowel functions are controlled by complex interactions between the voluntary and autonomic nervous systems. Following spinal cord injury, these interactions can become disrupted, causing overactivity in the bladder during the storage of urine, termed neurogenic detrusor overactivity (NDO). NDO leads to complications such as high bladder pressures, urinary incontinence, and potential kidney failure. The bowel similarly may experience involuntary contractions, often leading to fecal incontinence.

Current management therapies for bladder and bowel dysfunction include pharmaceutical and surgical techniques. Antimuscarinic medication (AM) such as oxybutynin, solifenacin, and tolterodine prevent incontinence by blocking the action of the neurotransmitter at the neuro‐muscular junction. However, AM is not specific to the bladder and often cause intolerable side effects such as blurred vision, constipation, and dry mouth. Surgical techniques include injections of the neurotoxin, onabotulinum toxin A, directly into the bladder wall to cause temporary paralysis. This technique requires costly repeat injections every 9–12 months and has on occasion caused systemic paralysis. More invasive and irreversible techniques include clam ileocystoplasty, the physical extension of the bladder. Each of these techniques can be effective; however, they can have significant side effects and still not effectively treat NDO. A 2010 study in the London Spinal Cord Injury Centre reported over half of the patients surveyed still experience regular incontinence [5].

Electrical stimulation (also known as neuromodulation) of the dorsal genital nerve, a sensory branch of the pudendal nerve, has been shown to effectively inhibit unwanted bladder contractions, thereby increasing capacity, improving continence, and reducing the potential for damage to kidneys [6, 7]. Dorsal Genital Nerve Stimulation (DGNS) may be applied noninvasively using simple sticker electrodes placed on the penis or clitoris and standard electrical stimulation waveforms. In addition to acute studies showing suppression of NDO, evaluation of this technique out of the clinic is limited to just six studies (including one of our own) with a total of 24 people with SCI [8, 9, 10, 11, 12, 13]. Results have been encouraging, but all studies have been relatively short (< 4 weeks), with inconsistent outcome measures used. No adverse effects have been reported, and each study has documented beneficial effects on urodynamic parameters (volume and pressure) and diary parameters including incontinence frequency; some have noted a carry‐over effect where baseline urodynamic parameters have improved over time. Authors have reported that participants were dissatisfied with system bulkiness and would like a more discreet system.

In recent work since 2015, we have shown that DGNS is more effective at reducing bladder pressures, increasing volume, and decreasing incontinence than other popular neuromodulation techniques such as tibial nerve stimulation and sacral nerve stimulation [14]. To highlight specific results, we have found DGNS to increase bladder capacity by 153 ± 146 mL or 117% ± 201% when participants were off any existing medication, or to increase bladder capacities by 105 ± 45 mL when used in addition to existing medication. In a 4‐week pilot study involving five participants with SCI, incontinence was reduced from 1.0 ± 0.5 to 0.1 ± 0.4 leaks per day while using DGNS [9].

This study investigated the feasibility of delivering DGNS in a home setting, using a wirelessly controlled stimulator, designed for the treatment of bladder and bowel incontinence. We measured the effects on bladder pressures and storage volumes during an 8‐week intervention. The aim was for the DGNS to provide a longer duration from initial sensation of urge to find an appropriate toilet, when used on top of existing antimuscarinic medication regimes and to investigate any carry‐over effects.

## 2 Methods

### 2.1 Device

The investigational device being used was the UCon Nerve Stimulator, manufactured by InnoCon Medical (Aalborg, Denmark), able to deliver current‐controlled stimulation up to 60 mA, at 200 μs and 20 Hz.

The cathodal electrode used was a silicone electrode, designed to improve adherence to irregular and moist surfaces, such as in the genital area (Patch Electrode, InncoCon Medical, Aalborg, Denmark) and the anode used was a 5 × 5 cm patch electrode (Axelgaard, PALS Platinum electrode) placed on the lower abdomen or inner thigh. The stimulation lead was attached by a magnet so that, if the lead was inadvertently pulled, this caused the magnet to detach (rather than the electrode) and triggered an alarm (device beeps), making reattachment more convenient. The stimulator was small enough to be stored in a pocket and stimulation could be applied by a pressing a button on the stimulator itself or wirelessly using a remote control. Stimulation was applied in one of two modes:

- Continuous stimulation: applied continuously under user's control.
- Urge stimulation: brief (60 s) stimulation on urgency.

### 2.2 Study Protocol

This trial was a single center, prospective, open‐label pilot study of DGNS, approved by the West Midland–Edgbaston Research Ethics Committee (REC Reference 22/WM/0155). The inclusion criteria were SCI (suprasacral, AIS A‐D); SCI sustained > 6 months ago; NDO; capable of using the device at home either independently or with existing support; willing and able to provide written informed consent. Exclusion criteria were as follows: recipient of intra‐detrusor botulinum‐A toxin injections within the last 6 months; previous surgical intervention on bladder/sphincters; showing positive leucocytes and nitrites on urinalysis on the day of investigation; pregnancy; cardiac pacemaker; active sepsis; history of significant autonomic dysreflexia with SCI.

**FIGURE 1:**
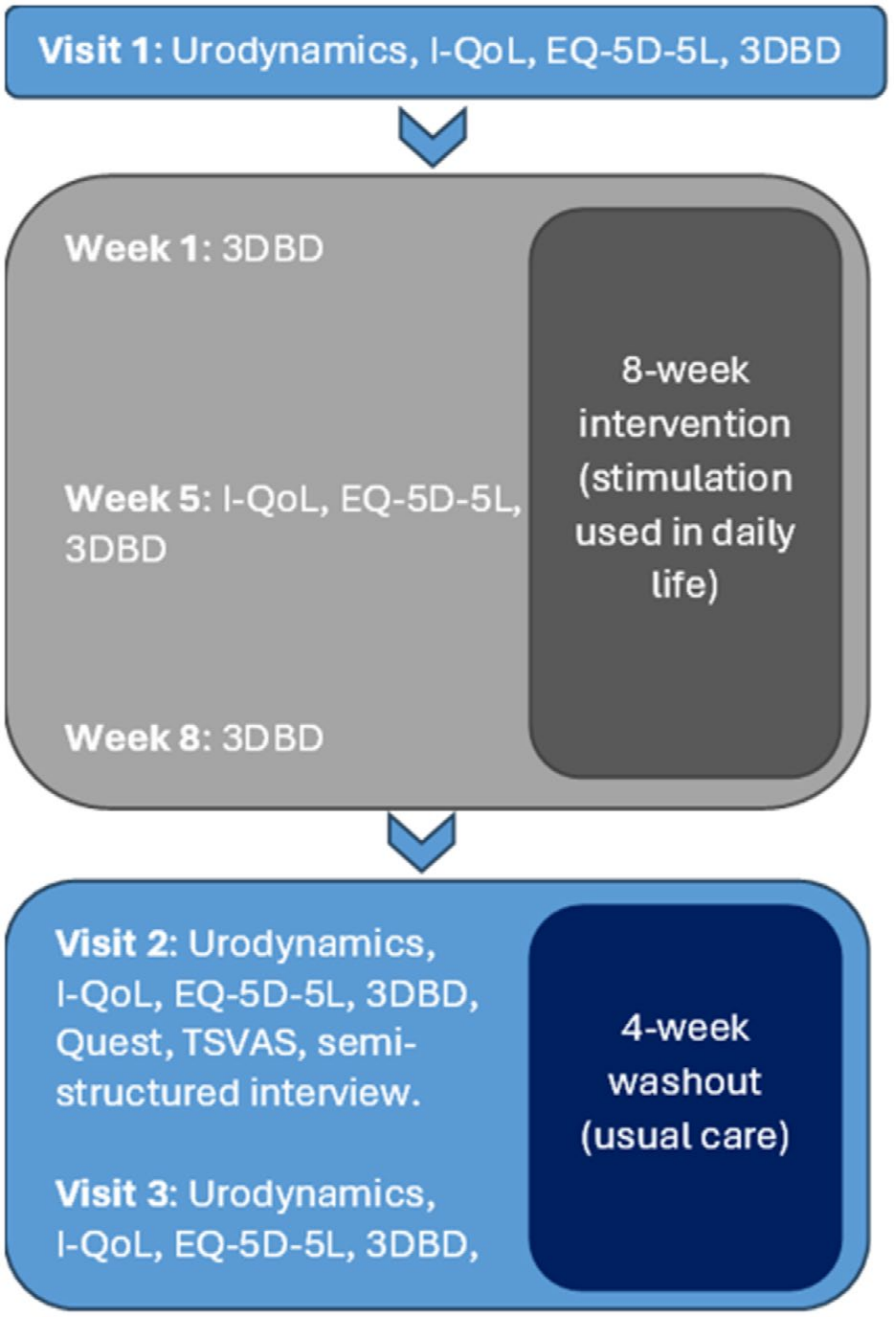
Flowchart of trial protocol. During visit 1, baseline outcome measures were completed, including urodynamics, Incontinence Quality of Life (I‐QoL) and EQ‐5D‐5L questionnaires and a 3‐day bladder diary (3DBD). Participants then used stimulation at home for 8 weeks and completed 3DBDs at 1, 4, and 8 weeks into the intervention. The I‐QoL and EQ‐5D‐5L were also completed at the intervention mid‐point. Baseline outcome measures were repeated at the end of the intervention and after a 4‐week washout. At the end of the intervention, participants also completed the Quebec User Evaluation of Satisfaction with Assistive Technology (Quest), Treatment Satisfaction Visual Analogue Scale (TSVAS), and a semistructured interview. [Color figure can be viewed at wileyonlinelibrary.com]

A flow chart of the trial protocol is shown in Figure 1. Recruited participants initially visited our laboratories at the Royal National Orthopaedic Hospital (RNOH) in Stanmore for a baseline urodynamic assessment with and without DGNS (see Section 2.2), a 3‐day bladder diary (3DBD), and completed baseline Quality of Life (QoL) questionnaires were recorded prior to use of DGNS. If NDO was present and could be suppressed by DGNS, the participant then used DGNS in their daily lives for 8 weeks, to control symptoms of NDO using the provided UCon stimulation system and patch electrodes. Participants with urge sensation used “urge stimulation” throughout the intervention period. Participants without urge sensation or with sensation but insufficient time to respond to it used “continuous stimulation” and were instructed to start the stimulation 2 h after they had last emptied their bladder.

During the 8‐week intervention, participants were asked to record three 3DBDs in the first, fourth, and eighth week of DGNS use. Each participant visited the RNOH following the 8‐week intervention to have urodynamics performed and to complete QoL and device acceptability questionnaires (see Section 2.6). Each participant then entered a 4‐week washout period, during which they did not use DGNS and continued with any existing regime. Participants recorded a 3DBD in the first and fourth weeks of washout. In the fourth week, participants also had a final urodynamics performed with and without DGNS and completed QoL questionnaires.

### 2.3 Urodynamic Assessments

Urodynamic assessments were performed at baseline, postintervention, and follow‐up by standard cytometry [15], which involves continuous monitoring of detrusor pressure by simultaneously measuring vesical and abdominal pressure during bladder filling. Two thin, or one dual lumen, urethral catheters were inserted into the bladder, one as a vesical pressure line and one as a line for filling and emptying. The bladder was initially emptied using a syringe and tested using a urinalysis dipstick to ensure no infection was present. A rectal balloon catheter was then placed in the anal canal to measure abdominal pressure. Transducers were zeroed to atmospheric pressure and located at the level of the symphysis pubis. The bladder was then filled retrogradely with room temperature, sterile saline solution at a rate of 30 mL/min. Detrusor pressure, urge rating, and fill volume were recorded until the end criteria were met (fill volume of 500 mL or urethral leakage or a sustained detrusor pressure > 40 cm H_2_O for 60 s or patient request/discomfort) when filling was stopped. Maximum cystometric capacity (MCC) and maximum detrusor pressure (MDP) were recorded when the end criteria were met. The participant's bladder was then emptied through the catheter, and the volume was measured using a measuring receptacle.

This filling and emptying process was then repeated four times, with a 5‐min break between the end of one fill and the beginning of the next, each using the same catheters and end criteria. During the second fill, DGNS was applied (see Section 2.3) when detrusor pressure increased by > 10 cm H_2_O. DGNS remained on for 60 s or until detrusor pressure decreased to < 10 cm H_2_O, whichever was longer, and was triggered again each time pressure increased until an end criterion was met. During the third fill, DGNS was applied by the participant on first sensation of urge, remained on for 60 s or until urge subsided, whichever was longer, and was triggered again each time a sensation of urge was felt until an end criterion was met. During the final fill, no DGNS was used. Catheters were then removed.

### 2.4 Dorsal Genital Nerve Stimulation (DGNS)

Self‐adhesive cutaneous stimulating electrodes (Innocon Medical) were placed over the dorsal genital nerve. For males, the cathode was positioned proximally on the dorsum of the penis. For females, the cathode was placed above the clitoris. The anode was placed on the abdomen or the inner thigh. During the baseline CMG assessment, stimulation was initially applied with biphasic pulses (200 μs pulse width) at 20 Hz, and stimulation intensity was progressively increased to determine sensory threshold and motor threshold for the pudendal‐anal reflex (PAR). Stimulation intensity was then set at 2 × PAR threshold or maximum tolerable intensity for the participant, whichever was lower.

During home use, stimulation was applied with biphasic pulses (200 μs pulse width) at 20 Hz at an intensity twice PAR threshold, as determined during the baseline CMG assessment, or the maximum tolerated by the participant, whichever was lower. Participants were given a program that enabled them to apply stimulation at this intensity at home, but they were able to adjust the intensity (within limits). We asked them to use the same intensity throughout the intervention, unless the sensation or effectiveness of the stimulation changed, in which case they could modify the intensity (within the set limits). Participants with retained bladder sensation applied stimulation conditionally: participants would trigger stimulation when they had a sensation of bladder fullness or the desire to void. For participants without sensation, stimulation was applied continuously between voids. Participants were requested to use the stimulation at least once per day.

### 2.5 3‐Day Bladder Diaries

Participants recorded standard 3‐day bladder diaries. Participants were asked to record fluid intake, voided volumes, leakage events, and urgency over 3 days on six occasions: prior to using the stimulator, for 3 days in the first, fourth, and final week during the 8‐week intervention, immediately after the intervention, and at the 4‐week follow‐up.

### 2.6 QoL Questionnaires

Participants completed two questionnaires; Incontinence Quality of Life (I‐QoL) and EQ‐5D‐5L at baseline, immediately after the intervention and after a 4‐week washout. The I‐QoL questionnaire assesses incontinence‐related QoL in three sub‐categories: “Avoidance & limiting behaviour,” “Psychosocial impacts,” and “Social Embarrassment.” A higher score indicates an improvement in QoL in each subcategory. The EQ‐5D‐5L questionnaire asks participants to rate how good or bad their health is today on a scale from 0 to 100 (0 means the worst health you can imagine and 100 means the best health you can imagine).

### 2.7 Device Acceptability

After the intervention, participants were asked to complete the Treatment Satisfaction Visual Analogue Scale (TSVAS) and the Quebec User Evaluation of Satisfaction with Assistive Technology (QUEST 2.0). The TSVAS asks participants to rate how satisfied they are with neuromodulation as a treatment for their neurogenic bladder dysfunction on a scale from 0 to 100 (0 means not satisfied at all and 100 means totally satisfied). The QUEST asks participants to rate how satisfied they were with various aspects of their assistive device (e.g., dimensions, securing and adjusting the device, durability, ease of use, comfort and effectiveness) on a scale from 1 to 5 (1 means not satisfied at all and 5 means very satisfied).

### 2.8 Semistructured Interview

At the end of the trial, a semi‐structured interview was also conducted to gain feedback on the participant's experience of using the device and participating in the trial. The interview was recorded and later transcribed.

### 2.9 Data Analysis

All data are presented as mean (±SD) unless stated otherwise. All data were tested for normality, and any data that were not normally distributed underwent log transformation. Two‐way analysis of variance (ANOVA) was used to compare MCC and MDP between control and DGNS fills and across timepoints (baseline, post‐intervention, and washout). One‐way ANOVAs were used to compare results from 3DBDs, I‐QoL, and EQ‐5D‐5L across timepoints. Interviews were transcribed, and themes were identified.

## 3 Results

### 3.1 Participants

**FIGURE 2:**
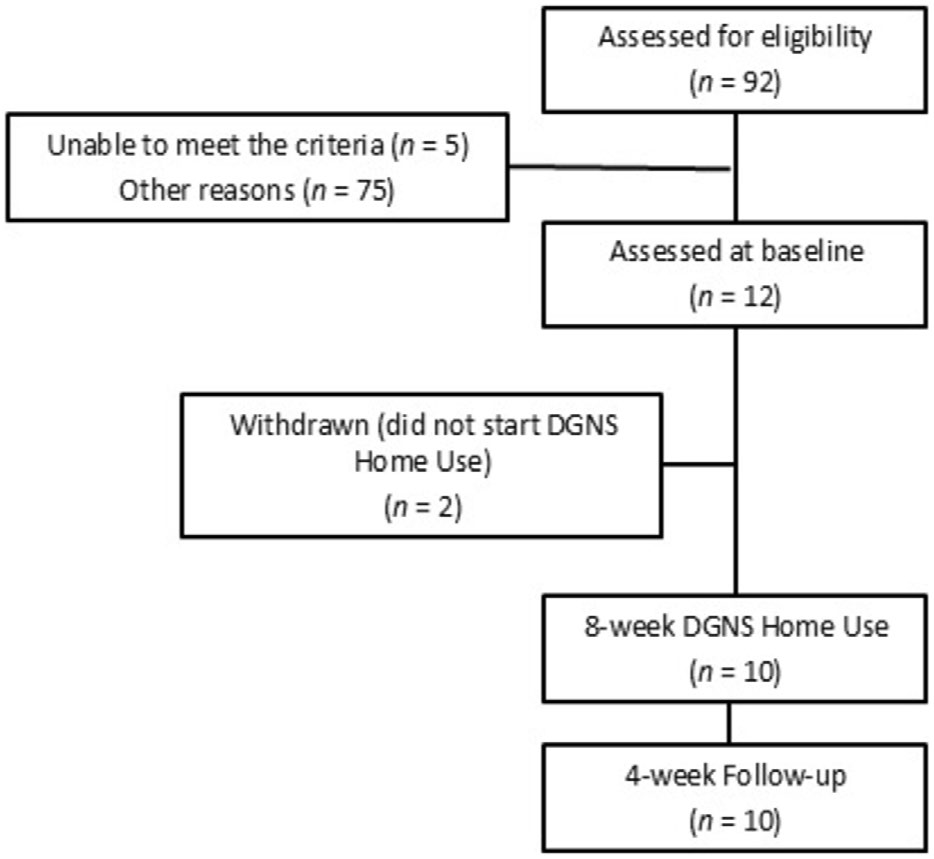
CONSORT flow diagram.

Twelve participants were recruited into the trial. A CONSORT flow diagram of participants in the trial is shown in Figure 2 and participant details are provided in Table 1. Ninety‐two participants were assessed for eligibility, and 12 were assessed at baseline. Two participants were withdrawn from the trial as they did not complete home use of DGNS for reasons unrelated to the investigational device. Ten participants completed the trial.

**TABLE 1:** Details of participants recruited into the trial.

ID	Age	Sex	BMI	SCI level	SCI AIS	Time since SCI	Storage management	Voiding management
NM01	55–59	M	25–29.9	L1	D	8		Urge + ISC
NM02	50–54	F	25–29.9	T12	B	3	Solifenacin 5 mg OD	Urge + ISC
NM03	40–44	F	18.5–24.9	T6	A	8	Trospium Chloride 60 mg OD, Mirabegron 50 mg OD	ISC
NM04	55–59	F	25–29.9	L1	D	1	Solifenacin 5 mg OD	ISC
NM05	65–69	M	25–29.9	T4	B	2	Trospium Chloride 60 mg OD, Mirabegron 50 mg OD	Urge + ISC
NM06	40–44	F	> 30	C5	B	1	Mirabegron 50 mg OD	SPC, clamps
NM07	35–39	M	18.5–24.9	T5	B	1	Solifenacin 10 mg OD	ISC
NM08	45–49	M	18.5–24.9	T6	B	19		Reflex voids, sheath
NM09	55–59	F	> 30	C6	B	1	Solifenacin 10 mg BD, Trospium 20 mg BD	SPC, clamps
NM10	45–49	M	> 30	C4	D	2	Solifenacin 10 mg OD	ISC
NM11	25–29	M	18.5–24.9	T4	A	23		ISC
NM12	55–59	F	18.5–24.9	C4	D	2		ISC

Abbreviations: AIS, ASIA impairment scale; BD, twice a day; F, female; ISC, intermittent self‐catheterization; M, males; OD, once a day; SCI, spinal cord injury; SPC, supra‐pubic catheterization.

### 3.2 UCon Device Use

Ten participants used the device at home for 8 weeks. PAR thresholds, determined during the baseline urodynamics session, stimulation intensity, and mode used by each participant at home are shown in Table 2. No adverse events were recorded in this trial. Two device deficiencies were reported; devices were replaced in both instances.

**TABLE 2:** Current intensity at pudendal anal reflex (PAR) threshold, current intensity used during CMGs at 0, 8, and 12 weeks and at home (mA), stimulation mode urge or continuous (cont) used at home and self‐reported usage at home for 10 participants.

Participant ID	PAR threshold	Stim amplitude for CMG			Stim amplitude for home‐use	Stim mode	Reported use
		0‐wk	8‐wk	12‐wk			
NM03	24	48	60	60	48	urge	Daytime everyday
NM04	36	36	36	24	36	urge	Daytime everyday except therapy
NM05	42	42	40	30	42	cont	Daytime everyday
NM06	21	45	48	48	45	urge	3 h everyday
NM07	25	48	48	48	48	cont	~5 times/week or when out of house, daytime
NM08	24	45	48	48	45	cont	3 h everyday
NM09	18	24	48	48	24	Urge	Daytime everyday except therapy
NM10	24	36	48	36	36	cont	Two 4‐h blocks, daytime and nighttime
NM11	36	60	60	60	60	cont	Daytime everyday
NM12	18	18	ND	42	18	cont	Daytime most days

### 3.3 Urodynamics

**FIGURE 3:**
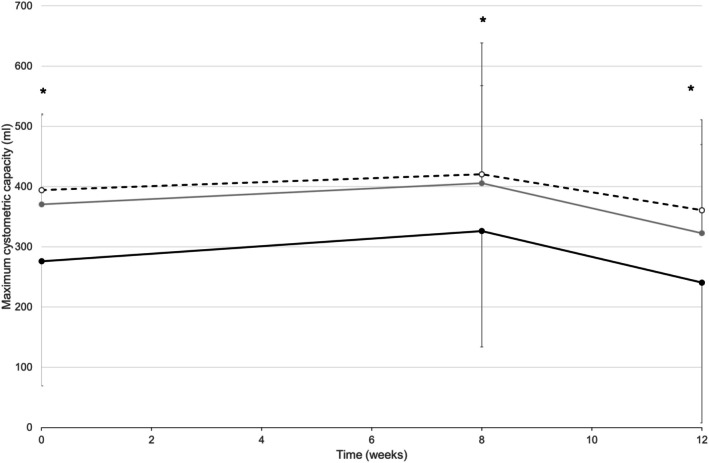
Average (SD) maximum cystometric capacity (MCC) measured during urodynamics at baseline (week 0), after the 8‐week intervention (week 8), and after the 4‐week washout (week 12). Results are presented for the initial control fill (solid black), fills with stimulation applied at onset of detrusor contraction (dashed black) and the final control fill (gray solid) from each urodynamics session. *Significant difference between the initial control fill and the fills with stimulation (*p* < 0.05).

During the preintervention, postintervention, and follow‐up urodynamics assessments, DGNS was applied at mean (SD) amplitudes of 40.0 (11.2), 47.8 (7.3), and 44.4 (11.1) mA, respectively. During the urodynamics assessment at preintervention, MCC significantly increased by 120 mL from 241 (105) mL in the initial control fill to 361 (140) during urodynamic fills with DGNS applied (*p* < 0.05; *N* = 10; Figure 3). Similarly, at the follow‐up assessment, MCC significantly increased by 118 mL from 276 (125) mL during the initial control fill to 394 (147) during urodynamic fills with DGNS applied (*p* < 0.001; *N* = 10; Figure 3). The increase in MCC from control fill to DGNS fills at the timepoint immediately after the intervention (8 weeks) was 94 mL and was approaching statistical significance (*p* = 0.056) due to greater variation in baseline data. MDP did not significantly change during urodynamic fills with DGNS applied compared with control fills at all timepoints (*p* < 0.05).

**FIGURE 4:**
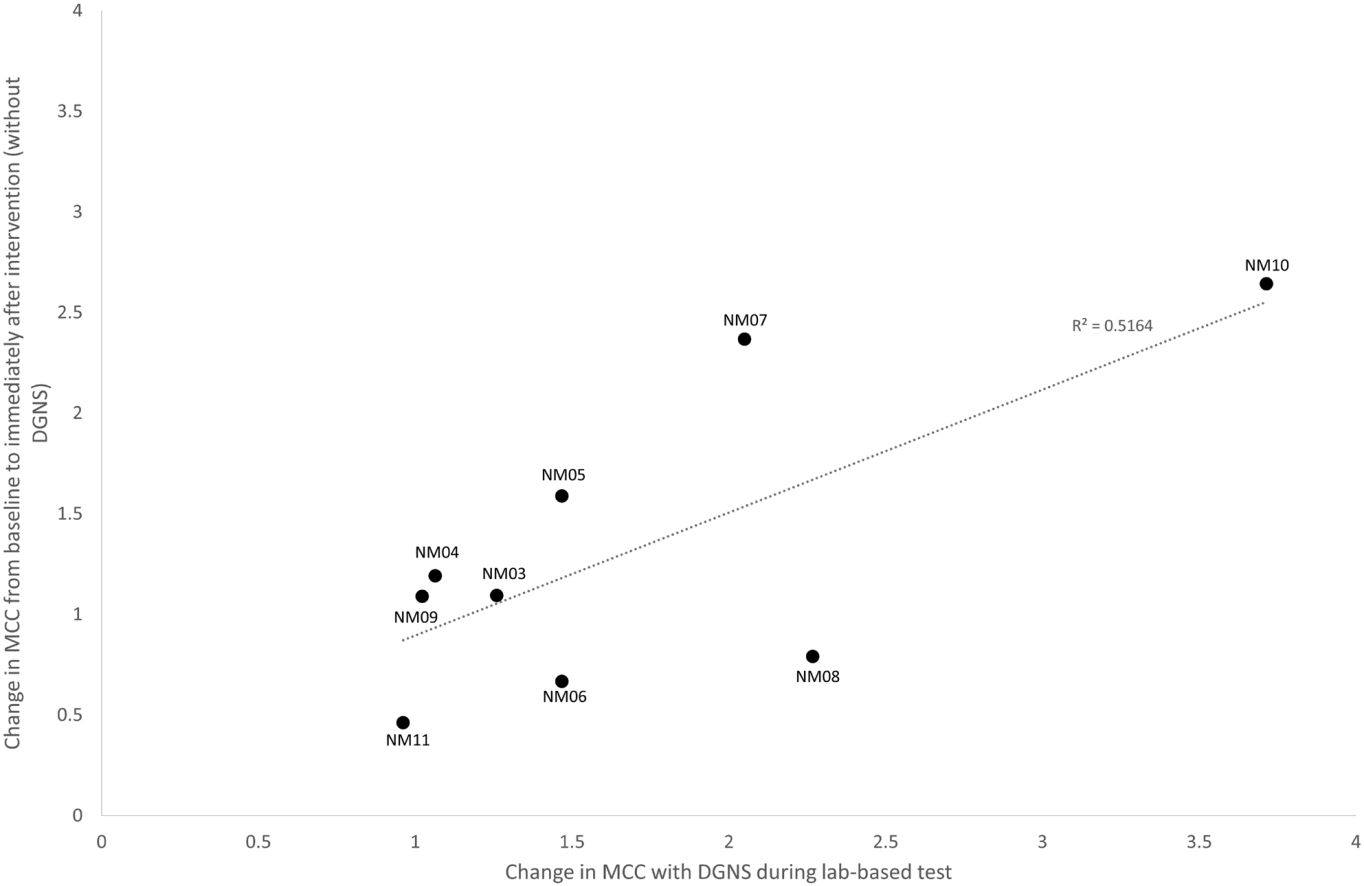
Change in maximum cystometric capacity (MCC) from the initial control fill to fills with DGNS at baseline plotted against the change in MCC from baseline to postintervention (without stimulation). Participants that responded better to the stimulation at baseline tended to show a greater improvement in MCC after the 8‐week intervention. Linear regression revealed a good correlation (R^2^ = 0.52).

During the urodynamics assessment at the end of the 8‐week intervention and at follow‐up, slight increases in MCC were noted compared with 0‐week during both the initial control fill and during fills with DGNS, but this was not statistically significant (*p* > 0.05). There was no correlation between the change in MCC from baseline to the end of the intervention and bladder capacity at baseline or stimulation intensity used at home (both absolute intensity and relative to pudendo‐anal reflex threshold). However, people that responded to DGNS well in the laboratory tended to respond better to DGNS used at home, although this was not true for all participants (Figure 4). There was also no significant change in MDP across timepoints (baseline, postintervention and 4‐week washout; *p* < 0.05).

### 3.4 3‐Day Bladder Diaries

**FIGURE 5:**
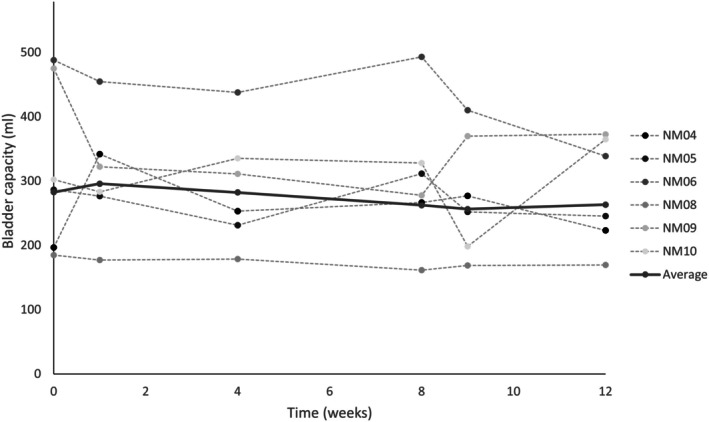
Average and individual bladder capacity from 3‐day bladder diaries (3DBDs), recorded at baseline (week 0), during the intervention (weeks 1, 4, and 8), post‐intervention (week 9), and after a 4‐week washout (week 12). Data are shown for the 6 participants that completed 3DBDs at all requested timepoints.

Bladder capacity was assessed by voided volumes reported in bladder diaries. Compliance with the completion of 3DBDs in this trial was relatively low. Six of 10 participants completed all six 3DBDs; however, the submitted diaries often did not include three full days of data. In some cases, participants with indwelling catheters did not always clamp them at baseline, resulting in high bladder volumes that were not reflective of true bladder capacity. Overall, no change in bladder capacity was observed from the 3DBDs during the 8‐week intervention and over the 4‐week follow‐up period (Figure 5).

From the 3DBDs, the reported number of incontinence episodes was 2.5 (3.8) at baseline. During the intervention, 1 (1.6), 1.7 (2.3), and 3.5 (4.3) incontinence episodes were reported during the first, middle, and final week. During follow‐up, 2 (3.3) and 2.3 (4.6) incontinence episodes were reported at 1‐ and 4‐week postintervention. No statistically significant changes in incontinence episodes were found (*p* > 0.05).

### 3.5 QoL Questionnaires

**FIGURE 6:**
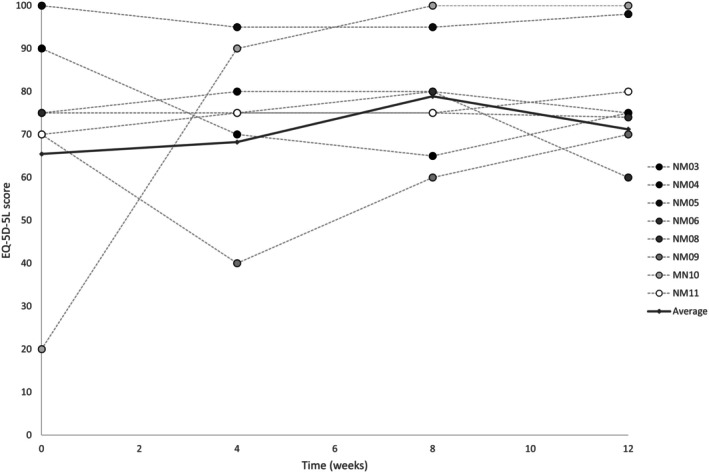
Average and individual EQ‐5D‐5L scores, recorded at baseline (week 0), during the intervention (week 4), postintervention (week 9), and after 4‐week washout (week 12). Data are shown for the eight participants that completed the EQ‐5D‐5L at all requested timepoints.

I‐QoL and EQ‐5D‐5L results are presented in Table 3 and Figure 6, respectively. Small overall improvements were noted in each I‐QoL sub‐category, most notably in “Avoidance & limiting behaviour,” which increased from 21.8 (6.0) at baseline to 24.4 (8.1) at the end of the intervention. Average (SD) EQ‐5D‐5L scores improved from 65.5 (25.0) at baseline to 78.9 (12.0) at the end of the intervention. One participant (NM10) reported a substantial improvement from 20 at baseline to 90 at 4 weeks and 100 at 8 weeks (end of intervention) and 12 weeks (end of 4‐week follow‐up).

**TABLE 3:** Individual and average (SD) Incontinence Quality of Life (IQoL) scores across three subcategories for 10 participants at baseline (0 weeks), intervention mid‐point (4 weeks), end of intervention (8 weeks), and follow‐up (12 weeks).

	Avoidance & limiting behavior				Psychosocial impacts				Social embarrassment			
Week	0	4	8	12	0	4	8	12	0	4	8	12
NM03	15	15	16	16	14	15	15	16	9	11	11	10
NM04	20	22	22	24	28	36	35	37	14	16	13	12
NM05	32	32	36	37	36	38	41	43	16	19	18	20
NM06	26	22	16	18	20	16	16	12	12	11	12	8
NM07	22	—	30	27	38	—	37	40	14	—	18	20
NM08	22	22	24	20	29	27	29	25	12	11	13	10
NM09	21	11	20	24	19	10	22	19	9	5	7	9
NM10	31	38	39	34	40	39	43	41	17	22	24	21
NM11	16	—	17	19	28	—	26	27	8	—	7	7
NM12	13	11	—	11	18	8	—	10	12	8	—	6
Average ± SD	21.8 ± 6.1	21.6 ± 9.0	24.4 ± 8.2	23.0 ± 7.6	27.0 ± 8.6	23.6 ± 12.1	29.3 ± 9.8	27.0 ± 11.9	12.3 ± 2.9	12.9 ± 5.3	13.7 ± 5.2	12.3 ± 5.9

### 3.6 Device Acceptability

The average (SD) device satisfaction score was 3.9 (0.8). A breakdown of average (SD) scores per question is provided in Table 4. Individual participant comments included that they could not find anywhere to fix the device to, that the electrodes were difficult to secure, and that the leads were fragile and often disconnected (e.g., during transfers). Nine participants completed the TSVAS after the 8‐week intervention period. The average (SD) TSVAS score was 79 (24), with 5/9 participants scoring > 90.

**TABLE 4:** Average (SD) Quebec User Evaluation of Satisfaction with Assistive Technology (QUEST 2.0) scores by question.

Assistive device how satisfied are you with	Average (SD) score
1. The dimensions (size, height, length, width) of your assistive device?	4.7 (0.7)
2. The weight of your assistive device?	4.8 (0.6)
3. The ease in adjusting (fixing, fastening) the parts of your assistive device?	3.3 (1.2)
4. How safe and secure your assistive device is?	3.4 (1.5)
5. The durability (endurance, resistance to wear) of your assistive device?	3.6 (1.1)
6. How easy it is to use your assistive device?	3.7 (1.4)
7. How comfortable your assistive device is?	4.4 (1.0)
8. How effective your assistive device is (the degree to which your device meets your needs)?	3.4 (1.6)

### 3.7 Semistructured Interview

The semistructured interview was conducted with nine participants. Considering the therapeutic effects of the DGNS, we identified six themes from the interviews.

#### 3.7.1 DGNS Use Within the Daily Routine

Participants fixed the device in place usually after morning care, and would wear it for 6–12 h, removing the device on return to bed. Three of the participants used the stim overnight, although two more participants wanted to use the stim at night. The main reason for not using the stim overnight was the need to charge the control unit and the risk of interrupted sleep due to the disconnection alarm.

NM04: “I wanted to use the stim overnight because of the ‘botherness’ [nocturia] at night but I'd rather charge the device for use tomorrow.”

NM03: “I wore the device in bed but the batteries don't last 24 h so it started beeping and woke me up.”

#### 3.7.2 Effect of the DGNS

Five of the participants said that the DGNS was effective in **increasing their bladder capacity**: “I did find it very effective. It works really well and by the end of the two months, I could see the volume increasing… I think initially, I was only getting volumes of about 200 and in the last few weeks, I was getting volumes of up to 600 mls” (NM06); **and suppressing their urge**: “It stops the urge and I didn't leak.” (NM09), “the principal thing was not to leak. I would wear a sheath in the early days and might go for four hours but would leak, whereas now, I would not expect to go [leak] within four hours” (NM05). The other four participants mentioned that DGNS did not make a difference to their bladder capacity or only made a small increase in bladder capacity: “I didn't really notice any less leakage per se maybe I drained 20‐30 ml more and there was not enough duration between the urgency of wanting to go to make me say it is more effective” (NM11).

Participants reported comfort in knowing that the device gave them longer to reach a toilet:

NM07: “So if I needed to pee, I kind of press the button for it to stimulate and it would give me a window to not have to rush to the toilet so I could take my time and actually get there.”

NM04: “Just extending the time that I needed, it gave me that extra time, a bit of a peace of mind, to find a toilet.”

NM10: “it seemed to help me very well because I seem to not empty my bladder so frequently.”

However, two participants reported that the effects on continence reduced soon after starting the intervention (NM08 and NM11).

NM08: “I got the urge to go, pressed the button, and I should go. And it did seem to work sometimes. I've used for so long then I'll start leaking. And so it sort of worked, but then it did not work.”

NM11: “I remember when we did the test for the first time. It showed some kind of promise but when I ended up using it, I didn't notice anything drastically different… so it was pretty much similar to how I was pre using it.”

Most participants reported that they would use the device in the long term due to improved continence.

#### 3.7.3 Other Therapeutic Effects

Participants noted additional beneficial effects to bowel management, lower limb spasticity, and sexual function:

NM03: “abdominal muscles are stimulated.”

NM10: “certain muscles in that pelvic floor may be stronger standing up…my bowels as well. They seem to have got a bit better recently.”

NM07: “but I find that it's actually helpful for my bowels and also my spasticity, really. It kills it [spasms] quite a lot.”

NM05: “When I touch myself here, where the jelly thing [cathode] would have been, I've never thought what it was, but it would very quickly lead to an erection.”

#### 3.7.4 Pain and Discomfort

Some participants reported that the stimulation caused discomfort. However, they reported that the discomfort was not too bothersome and the benefits to continence were greater than the discomfort:

NM09: “The first moment was absolutely terrible, but later, it's ok. The first moment is like it squeezes all of you.”

NM05: “No, I would not say it [stimulation] is comfortable. I got used to the pins and needles that came with it and it did seem to accentuate that… felt like nerves were reawakened.”

NM07: “The weird thing about it is that it actually felt good. It's like a different type of pain, coz I'm always in pain in terms of the spasms and stuff. So having this is a relief kind of pain and it's good for me. I liked using it.”

NM08: “First of all, when I first put it on, it might get uncomfortable. Get a spasm and then it wore off.”

NM04: “When it was painful, I did not turn it off. I just let the minute pass to get the effect.”

#### 3.7.5 Disconnection

Participants reported that when the therapy did not work, it was mostly due to a disconnection somewhere between the device and the skin. Disconnection triggered an alarm to sound on the device and a requirement to reattach, which caused social embarrassment. As a result, participants did not use the device when doing activities that were likely to cause disconnection (e.g., during exercise, transfers, walking, while sleeping at night).

NM03: “I found the connectors, the magnets, the cable ends both, you couldn't really secure them properly on both the silicon and the black patch. So any pronounced movement say I was leaning sideways to pick something off the floor, they would kinda rub against the skin and get disconnected… like transferring into the car they were getting disconnected… (when the device disconnected whilst at a meeting) everybody's looking at me because of the alarm and I can't say, excuse me, ‘I'm just going to put my hands under my trousers’.”

NM07: Talking about the gel electrode: “The only thing will be, because it can lift up so easily even though it is sticky, any type of fabric, it just lifts up and it just goes so I initially tried to put some micropore (tape) on top (of the gel electrode) and once the micropore is in place, stopping it from lifting up, it was fine.”

NM10: “The square [electrode] one stays on the skin better than the small one (gel electrode) which comes off at times. The small one easily disconnects from the skin but also you'll have to find a good place for it on your skin and when it stays, it works ok.”

#### 3.7.6 Bladder Management

Participants reported that they did not feel confident enough in the device to be able to change their normal bladder management routine.

NM08: When asked if his bladder management changed during the trial: “Not really, I suppose. So I was just trying it… Anyway, really couldn't rely on it.”

NM06: “I didn't feel confident to clamp outside the timed trial we'd actually use the device.”

## 4 Discussion

While dorsal genital nerve stimulation is well known to effectively suppress neurogenic detrusor overactivity in people with spinal cord injury in the laboratory setting [6, 7, 16, 17, 18], there are very little data on its longer‐term use in the community. This trial has investigated the clinical effectiveness and acceptability of a wearable DGNS device for bladder management in a home‐based trial lasting 8 weeks in people with chronic SCI and NDO while maintaining existing medication use. The device used for home‐based DGNS was small and allowed remote control of stimulation to improve usability in a home setting. The device was intended for bladder management and benefited from electrodes that had been designed for placement over the genital area. We found DGNS effectively increased maximum bladder capacity when tested in a laboratory setting and that home‐based DGNS was acceptable to participants. MCC did not significantly change after the intervention, and some participants benefited more than others from home‐based DGNS.

When tested in a laboratory setting, our trial supports several previous publications [6, 7], demonstrating significant increases in MCC from initial control fill to fills with DGNS during urodynamics. DGNS consistently (across all timepoints) increased MCC by ~100 mL, which is a clinically important increase in this population (> 80 mL) [19, 20]. After the 8‐week intervention, MCC had slightly increased during both control and DGNS fills compared to baseline, although this was not statistically significant. No significant change was found from the 3DBDs taken during the 8‐week intervention compared with baseline, which may be because several participants were not compliant in completing the 3DBDs. Participants were requested to complete six 3DBDs over the duration of the 13‐week trial. In future trials, the number of bladder diaries should be minimized.

It is possible that the potential improvement in bladder capacity was limited by the fact that DGNS was given in this trial as an adjunct to the participant's current bladder management. MCC at baseline was 276.0 (124.9) ml, with 5/10 participants having baseline bladder capacities > 300 mL, which is considered a clinically acceptable bladder capacity [7]. No correlation was found between baseline MCC and change in MCC from baseline to post‐intervention; nevertheless, we should consider being more selective in future trials by excluding people with baseline bladder capacity > 300 mL, as their bladder may already be effectively managed. A moderate correlation was noted between the change in MCC when DGNS was applied in the laboratory setting and change in MCC from baseline to post intervention (without DGNS). Therefore, response to DGNS in a laboratory‐based setting seems a useful parameter to indicate which people are most likely to benefit from home‐based DGNS, but we still need to understand the additional factors that contribute to variability in responders.

In this trial, we included participants with varying levels of preserved sensation in the genital area. We know from previous studies that the optimal stimulation amplitude for suppression of NDO is at least twice that required to elicit the pudendo‐anal reflex [7]. However, for some participants with intact sensations, it was not possible to reach this level of stimulation, due to discomfort, or the stimulation intensity could not reach the required intensity, particularly as the UCon device was limited to 60 mA output. The average current to elicit the pudendo‐anal reflex in the UCon study was 28.3 ± 8.5 mA and the average stimulation current used in the home setting was 47.7 ± 9.5 mA. Only five participants (NM3, NM6, NM7, NM8, and NM11) were able to use stimulation that was at twice the threshold current. However, no correlation was found between the intensity of stimulation used at home and change in MCC from baseline to post‐intervention.

The device used in this trial received high QUEST and TVAS scores, indicating that this form of intervention had a high acceptability. The main issues noted by participants related to the leads detaching frequently and difficulty in electrode placement (particularly in females). Despite the electrodes being designed for use in the genital area, they remained problematic, mainly due to the leads being pulled when moving around in daily life. Implantable neuromodulation devices remain the only option to overcome this problem. While implantable devices are available, many patients do not wish to undergo surgery.

It was clear that some participants benefited from the intervention substantially more than others. For example, participant NM10, who showed the highest response to DGNS in the laboratory and the greatest increase in MCC following the intervention, increased their I‐QoL score from 20 at baseline to between 90 and 100 at all timepoints during and after the intervention. Their bladder capacity increased based on 3DBDs and they additionally reported improvements in lower limb spasticity with the DGNS. NM10 purchased a commercially available stimulator after the end of the trial to enable them to continue DGNS use at home.

### 4.1 Limitations

The primary limitation of the study was that we were unable to stop pharmaceutical management of NDO in participants in the investigations due to safety concerns. This means that the DGNS was applied as an adjuvant therapy, and at this stage, we cannot determine how effective it would be in the absence of medication. Many patients experience side effects from antimuscarinic and alpha‐adrenergic medication, and being able to replace medication with DGNS would be advantageous. It should also be acknowledged that the number of subjects recruited into the trial was relatively low, and a control group was not included.

## 5 Conclusion

Dorsal genital nerve stimulation delivered through a wearable device is a potentially effective and well‐tolerated management technique for control of neurogenic detrusor over‐activity in people with spinal cord injury. This trial has demonstrated some positive effects of DGNS in people with SCI, with some participants substantially benefiting from the home‐based intervention. Improvements in the technology are still required to increase acceptability and a larger sample size is needed to assess the long‐term effects on bladder capacity.

## Author Contributions

Sean Doherty, Sarah Knight, Lynsey Duffell, Richard Nobrega: concept/design; Sean Doherty, Evangeline Martinez, Sarah Knight, Lynsey Duffell: data analysis/interpretation; Sean Doherty, Evangeline Martinez, Sarah Knight, Lynsey Duffell: drafting article; Sean Doherty, Evangeline Martinez, Sarah Knight, Lynsey Duffell, Richard Nobrega: critical revision of article; Sean Doherty, Evangeline Martinez, Sarah Knight, Lynsey Duffell, Richard Nobrega: approval of article; Lynsey Duffell: statistics; Sean Doherty, Sarah Knight, Lynsey Duffell: funding secured by; Sean Doherty, Evangeline Martinez, Sarah Knight, Lynsey Duffell: data collection.

## Conflicts of Interest

The authors declare no conflicts of interest.
